# LZerD webserver for pairwise and multiple protein–protein docking

**DOI:** 10.1093/nar/gkab336

**Published:** 2021-05-08

**Authors:** Charles Christoffer, Siyang Chen, Vijay Bharadwaj, Tunde Aderinwale, Vidhur Kumar, Matin Hormati, Daisuke Kihara

**Affiliations:** Department of Computer Science, Purdue University, West Lafayette, IN 47907, USA; Department of Computer Science, Purdue University, West Lafayette, IN 47907, USA; Department of Computer Science, Purdue University, West Lafayette, IN 47907, USA; Department of Computer Science, Purdue University, West Lafayette, IN 47907, USA; Department of Computer Science, Purdue University, West Lafayette, IN 47907, USA; Department of Computer Science, Purdue University, West Lafayette, IN 47907, USA; Department of Computer Science, Purdue University, West Lafayette, IN 47907, USA; Department of Biological Sciences, Purdue University, West Lafayette IN, 47907, USA; Purdue University Center for Cancer Research, Purdue University, West Lafayette, IN 47907, USA

## Abstract

Protein complexes are involved in many important processes in living cells. To understand the mechanisms of these processes, it is necessary to solve the 3D structures of the protein complexes. When protein complex structures have not yet been determined by experiment, protein-protein docking tools can be used to computationally model the structures of these complexes. Here, we present a webserver which provides access to LZerD and Multi-LZerD protein docking tools. The protocol provided by the server have performed consistently among the top in the CAPRI blind evaluation. LZerD docks pairs of structures, while Multi-LZerD can dock three or more structures simultaneously. LZerD uses a soft protein surface representation with 3D Zernike descriptors and explores the binding pose space using geometric hashing. Multi-LZerD performs multi-chain docking by combining pairwise solutions by LZerD. Both methods output full-atom docked models of the input proteins. Users can also input distance constraints between interacting or non-interacting residues as well as residues that locate at the interface or far from the interface. The webserver is equipped with a user-friendly panel that visualizes the distribution and structures of binding poses of top scoring models. The LZerD webserver is available at https://lzerd.kiharalab.org.

## Graphical Abstract

**Graphical Abstract:**
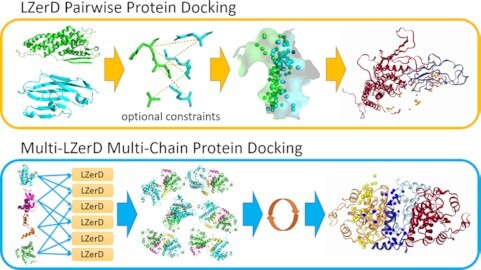
The LZerD Protein Docking Server provides pairwise docking with LZerD and multi-chain docking with Multi-LZerD.

## INTRODUCTION

Protein complexes are central to many processes in a living cell. To understand the physical mechanisms of these processes, determining the 3D structures of their associated protein complexes is a critical step. When protein complex structures have not yet been determined by experiment, it is possible to use computational tools to construct models of these complexes (1). A protein docking program can take two or more component protein structures as input and assemble them into atomic structure models of the protein complex. Here, we present a webserver which provides easy, installation-free access to the LZerD software suite, including LZerD (2) for pairwise protein docking and Multi-LZerD (3) for docking three or more proteins simultaneously.

There are several protein-protein docking methods and their derivatives are publicly available, such as ZDOCK (4), HADDOCK (5), ClusPro (6), RosettaDock (7), HEX (8), SwarmDock (9) and ATTRACT (10). Compared to other docking methods, the LZerD docking web server is unique in the following aspects: First, the Multi-LZerD functionality available on our server is unique in that it facilitates global multi-chain docking search. This modeling is not restricted to symmetrical docking. Multi-LZerD supports modeling of general asymmetric multi-chain complexes. Second, generated models are ranked by a scoring system that is consistently ranked among top in CAPRI (11). Third, users can specify interacting and non-interacting residue constraints in a flexible fashion by specifying a distance range for each constraint. Fourth, the distribution of docking poses of generated decoys is easily understood by an intuitive visualization panel.

In addition to this capability, The LZerD suite has been consistently ranked highly in the server category in CAPRI (11), the blind communitywide assessment of protein docking methods. By top-1 model quality, LZerD ranked top among servers in CAPRI rounds 38–45 during 2016–2018 for both docking prediction and scoring (11). By the same measure in CAPRI round 46, a joint round with CASP13, LZerD ranked second for docking prediction and top for scoring (12). The LZerD webserver makes this automated docking capability available for easy use without any installation on the part of the end user, and without a requirement that end users have any direct access to high-performance computing resources.

## MATERIALS AND METHODS

The LZerD webserver presents a convenient interface to the LZerD suite of protein docking tools at https://lzerd.kiharalab.org. Currently, LZerD and Multi-LZerD interfaces are made available. The LZerD (Local 3D Zernike descriptor-based docking) algorithm handles pairwise docking of protein structures (2). First proteins are converted into a soft surface representation using 3D Zernike descriptors (3DZDs) (13). Then, using 3DZDs and other molecular surface features, sites on each input protein's surface are matched together to generate docked conformations. 3DZDs realize a soft surface representation that tolerates some protein flexibility of main- and side-chains up to a couple of angstroms of deviation that do not affect surface shape much (2).

Models are clustered using a user-defined cutoff and scored as described in later sections. Multi-LZerD (3) is a generalization of LZerD which can dock more than two proteins simultaneously. Multi-LZerD combines subunit pairs docked by LZerD using a genetic algorithm with a molecular mechanics-based scoring function, resulting in a stochastic search of the multi-subunit conformational space. By mutating a population of docked models and retaining only the best-scoring members over the course of thousands of iterations, the genetic algorithm generates full-complex models which agree with the pairwise docking results both individually and taken together.

### General workflow

**Figure 1.**
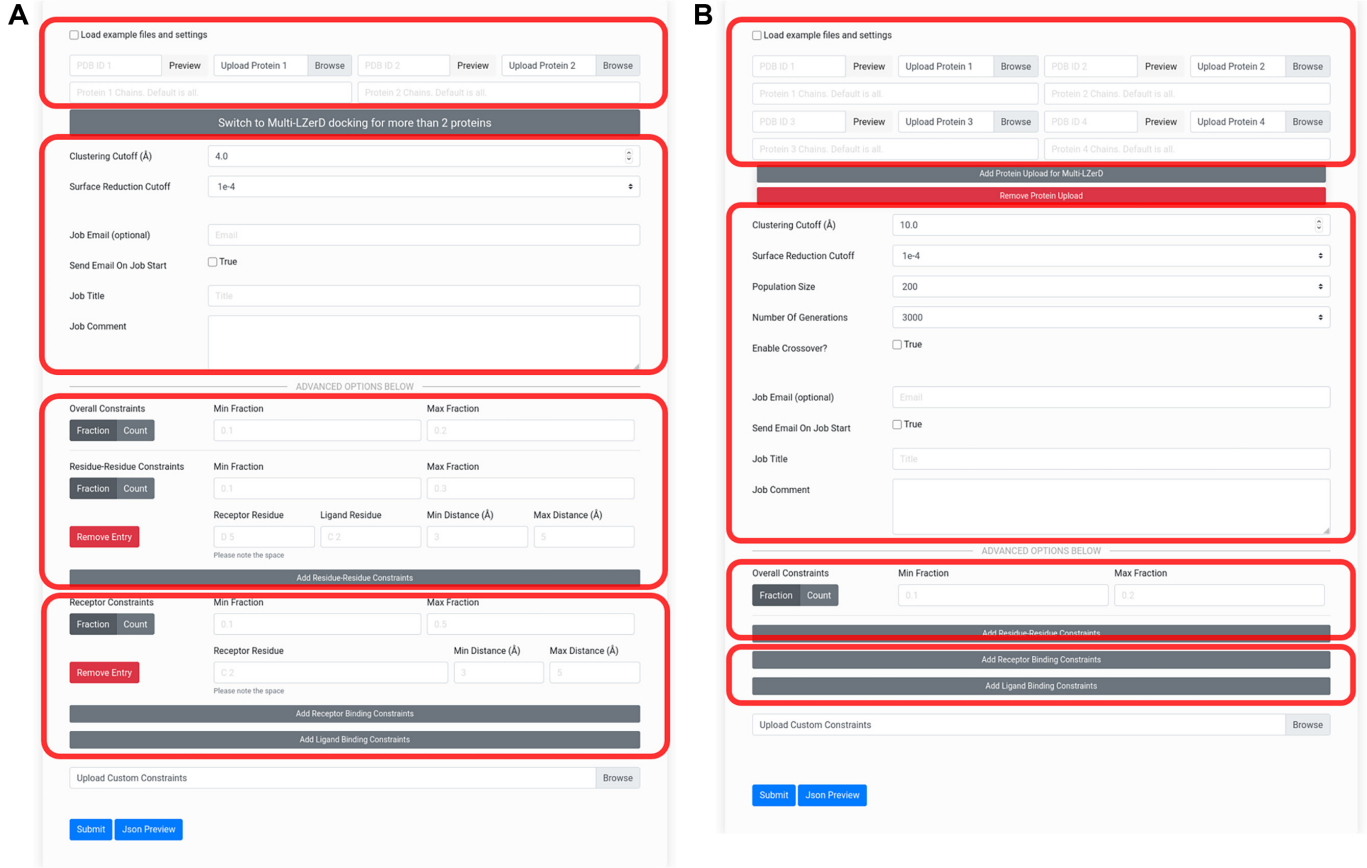
Docking job submission interfaces. (**A**) The submission form for pairwise LZerD protein–protein docking jobs. The first area outlined in red is for specifying input subunit structures: the toggle allows users to quickly load example subunit structures and constraints, while the file upload buttons allow users to upload their own structures. Users can alternatively specify an entry to fetch directly from the PDB. Chain IDs to extract from the individual subunits can be entered. When a structure is loaded, a 3D interactive preview is displayed. Then, there is a button to switch to the Multi-LZerD form. The second area is for specifying general job parameters: the clustering RMSD cutoff controls the structural redundancy of the output models (higher RMSD means more diverse output structures), the surface reduction cutoff controls the fineness of the conformational sampling (lower means finer sampling), and optional user email and job title and comment settings control notifications and annotations. In the third and fourth areas, in the Advanced Options section, residue-residue or binding site constraints, respectively, can be added by clicking the Add buttons and filling in the residue number(s) and distance range fields. Users can toggle between min/max fraction and min/max count of constraints that must be satisfied. For more details, check the How to Use page on the server. (**B**) The submission form for Multi-LZerD multiple docking jobs involving 3–6 proteins. The Multi-LZerD form has Add and Remove buttons below the file upload buttons, which can be used to add or remove subunit structures. Any number of subunit structures from 3 to 6 may be uploaded. The areas outlined in red correspond to the outlined areas of the LZerD submission form.

It is highly recommended that users register (https://lzerd.kiharalab.org/register/) as a first step. Neither registration nor an email address is required to submit jobs; however, there are productivity benefits to registration. Jobs not associated with any account may not be retained longer than two weeks, while jobs from registered users are retained for at least three months. Furthermore, registered users can view a table of retained jobs on their account, allowing for easier management of their results. Regardless of whether a user is registered, users can submit jobs for the different docking methods using their respective submission pages (Figure 1).

For both LZerD and Multi-LZerD, the general procedure is essentially the same. First, the user uploads their subunit structures to dock; currently, structures in the PDB format are accepted. Users can use experimentally determined structures if available or computationally modeled structures of subunits. Alternatively, the user can specify a PDB entry to use. Specific chains can be chosen by ID to use as the subunit. Then, algorithm parameters such as clustering cutoff can be adjusted from their default settings as the user desires. If the user is registered and logged in, their email address will be automatically filled in. Non-registered users may optionally fill in their email address. The user can annotate their job with a title and/or comment for their own organizational purposes; job titles are listed in the job tracking table for registered users. Finally, users can fill in or upload any desired residue-based distance constraints in the advanced options section. Specifying constraints is not required. Users can provide contacting or non-contacting residue pairs by specifying their allowed distance ranges or can simply specify residues which are at the docking interface or which are far from the interface. As demonstrated in Discussion, LZerD suite tools can produce accurately assembled complex models de novo for many cases. However, the inclusion of such constraints properly derived from experimental data can improve even already-accurate docked models.

Once all desired settings are configured, clicking Submit will queue the job for processing. Job completion time will vary by structure size and docking method, as discussed below. Upon successful job submission, the user is provided with a link that will display the status of the job. Once the job is completed, the link will show the results. If an email address was provided, the user will be notified by email when their job completes. Users may also opt to be notified when their job begins running.

For all the docking methods currently available through the webserver, the final output models are scored using a rank aggregation procedure called ranksum. Ranksum uses the sum of model ranks by the knowledge-based scoring functions DFIRE (14), GOAP (15), and ITScorePro (16) to select models by a consensus of these scoring functions. Ranksum has been demonstrated effective in several rounds of the CAPRI blind community-wide experiment, where it was used to achieve top performance in scoring models (11,17). In the webserver output we provide the model ranking by ranksum as well as ranks by the individual scores as reference. Users are encouraged to primarily pay attention to ranksum results for selecting models.

### Using the LZerD via the web interface

To assemble two protein structures, users should use LZerD (https://lzerd.kiharalab.org/upload/upload/). Once the user has uploaded their two structures, here referred to as the receptor and the ligand, based on the order they were submitted in, they can configure any desired custom settings. Figure 1A shows the LZerD submission page. The default settings are recommended, but users can change the clustering cutoff, which controls the amount of redundancy allowed among the output models, and the surface reduction cutoff, which controls the fineness of the sampling of the conformational space. Using a higher surface reduction cutoff can result in faster docking at the expense of accuracy.

In the advanced options section, users can specify residue-based distance restraints that are known from available experimental evidence from the literature. Computational predictions can be also used to obtain residue constraints (18,19). Residue-residue constraints specify the allowed distance range between two residues, where distance is defined by the closest heavy (non-hydrogen) atom pair between those residues. Receptor binding site constraints specify the allowed distance range between a receptor residue and the nearest ligand heavy atom. Ligand binding site constraints are similar but consider a ligand residue and the nearest receptor heavy atom. For example, a contacting residue pair may be specified with a distance range of 3–5 Å. On the other hand, if a residue pair should not be in contact, a substantial minimum distance between them can be specified, e.g. 10 Å. Similarly, residues at the interface can be specified with a distance of 3 to 5 Å (without specifying an interacting partner), whereas non-interacting residues can be described with a distance of 10 Å or higher. Moreover, using the min/max fraction fields, users can control what proportion of these constrains must be satisfied for a model to be included in the output. This can be switched from fraction mode to count mode, where the user instead explicitly specifies the numbers of constraints which should be satisfied.

**Figure 2.**
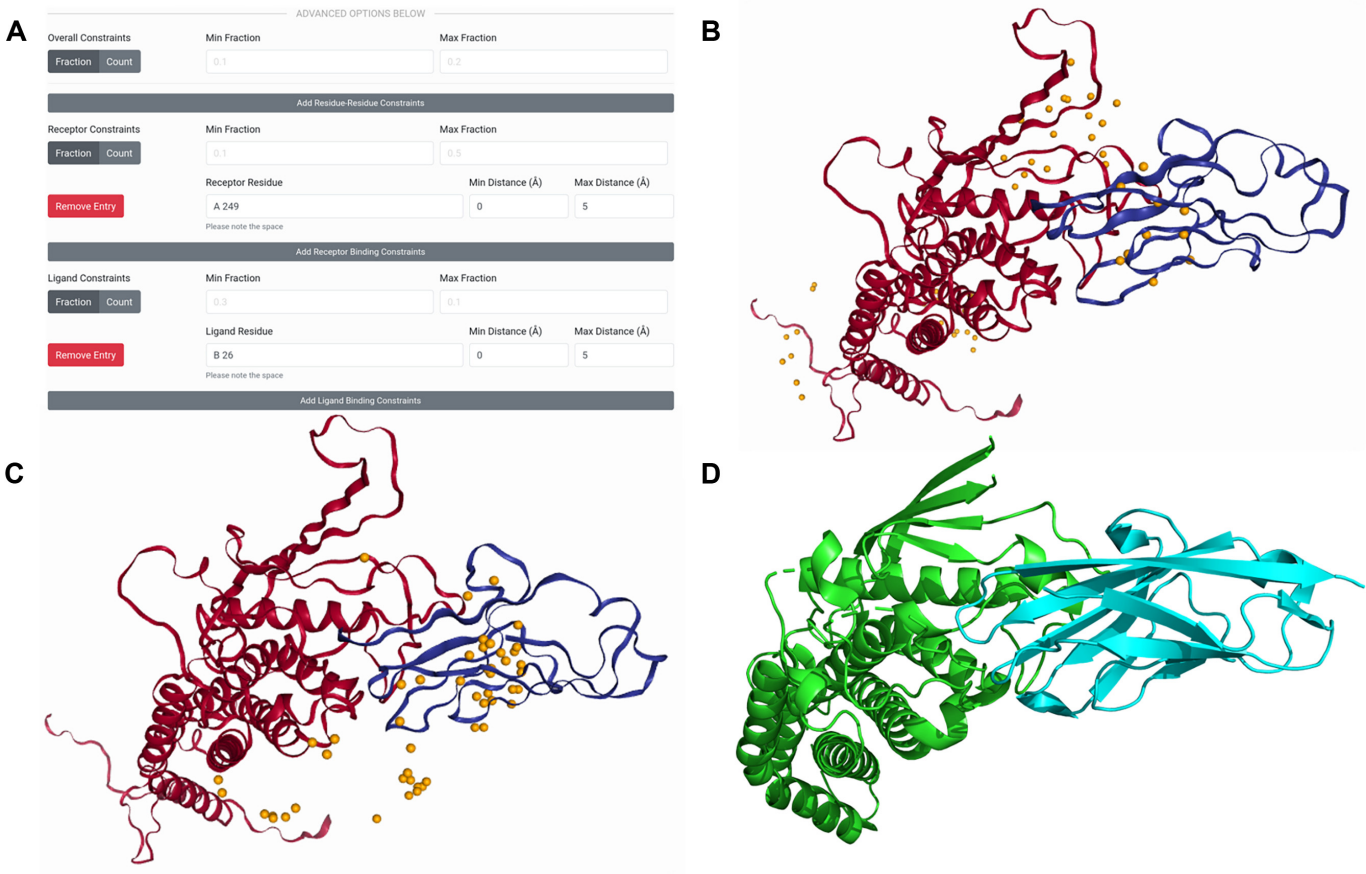
Docking input and results for HopQ-II:CEACAM1 pairwise docking. The template-based models for HopQ-II and CEACAM1 are shown in red and blue respectively in their best-scoring docked conformations. The native complex structure is shown likewise in green and cyan. (**A**) The constraints section of the LZerD submission form with the receptor and ligand binding sites for the constrained docking filled in. Here, receptor model chain A residue 249 is specified to be in the range of 0 to 5 Å from the nearest ligand heavy atom. Also, ligand model chain B residue 26 is specified to be in the range of 0 to 5 Å from the nearest receptor heavy atom. The min/max fraction fields are blank, leaving the default that all constraints must be satisfied simultaneously. (**B**) The results of docking without constraints. The centroids of the top 50 models by ranksum are shown as orange spheres. The top model shown has an I-RMSD of 3.1 Å and an ${f_{nat}}$ of 0.39, which is considered of acceptable quality under the CAPRI criteria. (**C**) The results of docking with the constraints shown in A. the centroids of the top 50 models by ranksum are shown as orange spheres. The top model shown has an I-RMSD of 2.6 Å and an ${f_{nat}}$ of 0.41, which is considered of medium quality under the CAPRI criteria. (**D**) The native structure of the complex (PDB ID: 6GBH), shown with the receptor (HopQ-II) superimposed to the same orientation as in the docked models.

Once docking is completed, the results are presented in visual and downloadable form. The main feature of the LZerD results page is the 3D visualization (see Figure 2B, C). In the default mode, the distribution of ligand docking conformations around the receptor is shown by spheres representing the centroid of the top 50 models. Hovering over a sphere shows a tooltip with the model rank, and clicking it displays the full model. Users can change the number of centroids shown and the model representation and can also download the docking output in PDB format. Below the 3D structure panel, the ranksum results and individual component scores for each model is shown. Individual models can be loaded into the 3D structure pane by clicking and can also be downloaded. Many models, top 10, 50, 500 scoring models or all the generated models of over 50 000 can be also downloaded at once as a zipped file from the links at the top of the result page.

### Using the Multi-LZerD web interface

**Figure 3.**
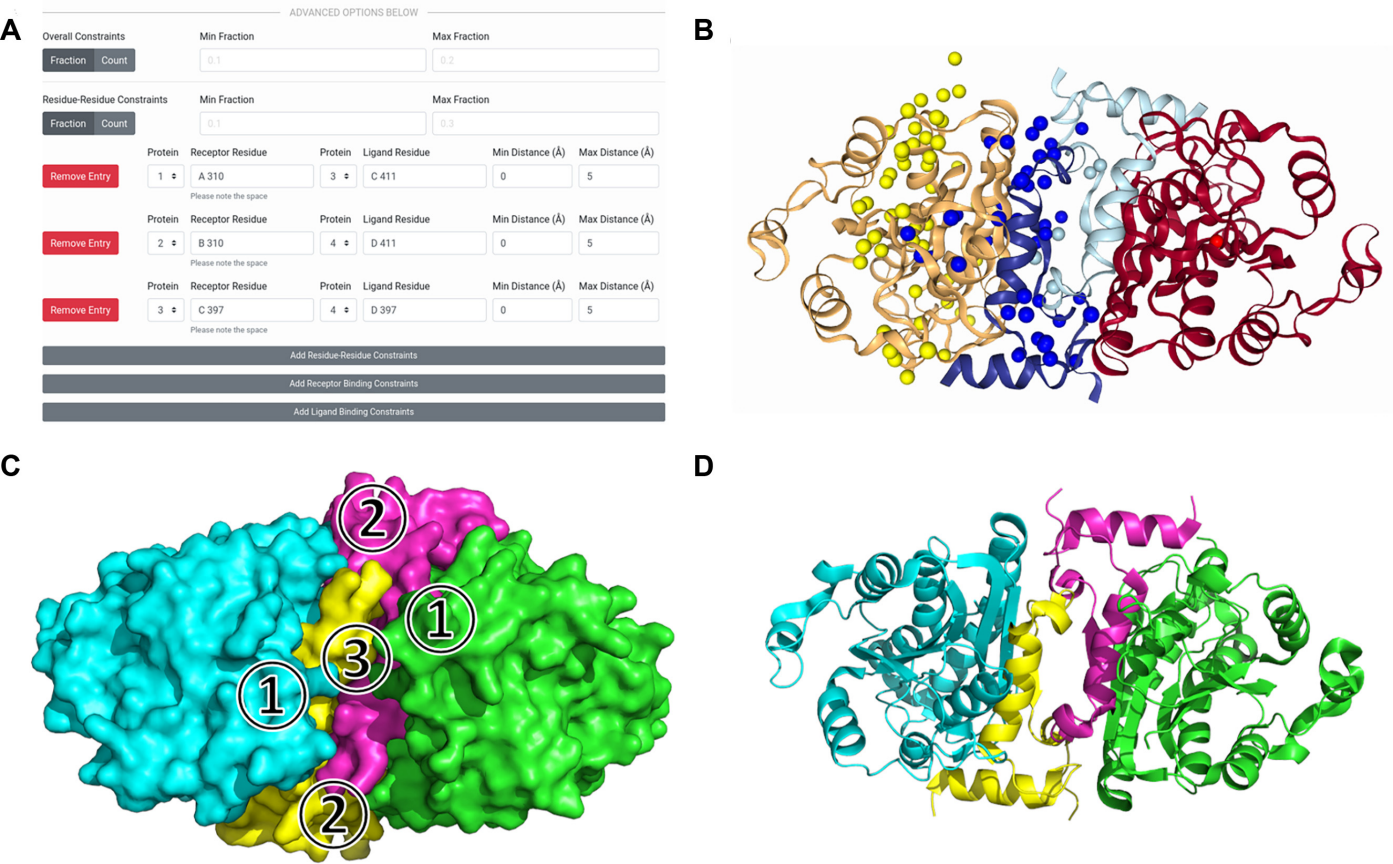
Docking input and results for enoyl-ACP reductase multiple docking. This complex has four chains of size between 60 and 229 amino acids. (**A**) The constraints section of the Multi-LZerD submission form with the residue-residue interaction information to be integrated filled in. Here, distances in the range of 0 to 5 Å are specified for each of the pairs: subunit 1 chain A residue 310 and subunit 3 chain C residue 411, subunit 2 chain B residue 310 and subunit 4 chain D residue 411, and subunit 3 chain C residue 397 and subunit 4 chain D residue 397. The min/max fraction fields are blank, leaving the default that all constraints must be satisfied simultaneously. (**B**) The results of docking. The centroids of the top 50 models by ranksum are shown as spheres and are colored according to which subunit they represent. (**C**) A diagram of the protein-protein interfaces. In the top model shown in B, 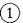
has an I-RMSD of 1.05 Å and an ${f_{nat}}$ of 0.79, 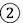
has an I-RMSD of 1.38 Å and an ${f_{nat}}$ of 0.91, and 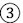
has an I-RMSD of 1.02 and an ${f_{nat}}$ of 0.80. All three unique interfaces are modeled to medium quality under the CAPRI criteria. (**D**) The native structure of the complex (PDB 1NNU), shown superimposed to the same orientation as the docked models.

To assemble three or more proteins, users should use Multi-LZerD (https://lzerd.kiharalab.org/upload/MultiLZeRD/). Via the web interface, up to six subunits can be submitted for a docking job. After using the Add button to set the appropriate number of subunits, users can upload their structures just like for LZerD in the previous section (see Figure 1B). In addition to the user-configurable parameters inherited from pairwise LZerD and described in the previous section, users can also set the population size (higher population explores more of the search space) and the number of generations (iteration limit) for the genetic algorithm optimization. As with pairwise LZerD, users should typically keep the default settings. Multi-LZerD also support constraints using the same interface as LZerD, with the addition of a field for specifying which subunit a residue belongs to. Once docking is completed, the results are shown using the same visualization as pairwise LZerD, but with the centroids color-coded for each subunit (see Figure 3B).

## DISCUSSION

To illustrate the usage and capabilities of the LZerD webserver, we present case studies showing how the features of the webserver can be used to generate accurately docked models of protein complexes.

### Case study: using LZerD to model the HopQ-II:CEACAM1 complex

The LZerD web interface can be used to recreate part of the LZerD server group's top performance in modeling T132 from CAPRI round 42 (11). T132 is a complex of a bacterial protein HopQ-II with a human host protein CEACAM1, the structure of which was not public during CAPRI round 42. The input structures used here are as for the LZerD server group during CAPRI: a template-based model of HopQ-II based on PDB 5LP2, and a template-based model of CEACAM1 based on PDB 5F1D, both built using MODELLER (20). The root-mean-square deviation (RMSD) of the computational models of the two subunits were 2.8 and 1.0 Å, respectively. As borne out by the docking performance, these subunits were individually well-modeled, which is important for assembly by free docking. Initially, we submit a job with no constraints, as was done in CAPRI. Figure 2B shows the centroid distribution and top model for this docking run. Even without any refinement, this model is acceptable under the CAPRI criteria, with an interface RMSD (I-RMSD) of 3.1 Å and a fraction of native contacts (${f_{nat}})$ of 0.39 relative to the native structure PDB 6GBH shown in Figure 2D. The top 10 models from this result in fact contain two such acceptable models, but it is clear from the distribution of centroids that the output contains many (albeit lower-scored) models far from the native binding site.

We can focus the modeling by supplying extra information via constraints. Again we submit the same subunit models, but additionally specify a receptor binding site residue and a ligand binding site residue as shown in Figure 2A. On the HopQ-II side, we specified that Asn264 (residue 249 in the template-based model), which replaces part of a hydrophobic platform found in HopQ-I (21), should be between 0 and 5 Å from the nearest CEACAM1 heavy atom. On the CEACAM1 side, we specified that Phe29 (residue 26 in the template-based model), which is implicated in the mechanics of specificity between different HopQ and CEACAM types (21), should likewise be between 0 and 5 Å from the nearest HopQ-II heavy atom. We leave the min and max fraction fields blank, which as a default means both of our constraints will be satisfied. In practice, residues to constrain can be identified from the literature or from experiment. In the docking result shown in Figure 2C, the docking has been clearly focused around the native binding site. In fact, the top model is now of medium CAPRI quality, with an I-RMSD of 2.6 Å and an ${f_{nat}}$ of 0.41. Thus, using the web interface it is straightforward to replicate the pre-refinement performance of the LZerD CAPRI server group. The computation for docking this target took less than one hour.

### Case study: using Multi-LZerD to model the enoyl-ACP reductase complex

Using Multi-LZerD, we can take on an expanded range of modeling tasks dealing with more than two protein subunits at a time. Here we demonstrate by modeling the assembly of four chains of a malarial enoyl-ACP reductase complex. While pairwise LZerD can often assemble a protein complex without the need constraints, the conformational search space Multi-LZerD must search is far larger. Thus, for performance, it is recommended that constraints be supplied when using Multi-LZerD. Here, we supplied residue-residue contact constraints as shown in Figure 3A. We selected highly conserved hydrophobic residues for the putative contacting pairs (22). Between chains A and C (and identically B and D), we specified Ile310 and Ile411. Between chains C and D, we specified Phe397 on both sides. For all these constraints, we specified the allowed distance range between their nearest heavy atoms as 0–5 Å. We leave the min and max fraction fields blank, which as a default means all three of our constraints will be satisfied. We then uploaded subunit models generated by SWISS-MODEL (23) as in (3). Figure 3B shows the centroid distribution and the top model for this docking run. This complex was modelled well overall, with a global RMSD to the native structure of 2.06 Å. In the native structure of this complex (see Figure 3D), there are three unique interfaces (see Figure 3C). Interface 1 is between chains A and C, and identically between chains B and D; interface 2 is between chains A and D, and identically between chains B and C; interface 3 is between chains C and D. Following the way such modeling is evaluated in CAPRI, we examine the accuracy of each distinct interface individually. In the top model returned by Multi-LZerD, the A:C interface is of medium quality under the CAPRI criteria, with an I-RMSD of 1.05 Å and an ${f_{nat}}$ of 0.79; the A:D interface is of medium quality under the CAPRI criteria with an I-RMSD of 1.38 Å and an ${f_{nat}}$ of 0.91, even though no constraints were placed on this interface; the C:D interface is of medium quality under the CAPRI criteria with an I-RMSD of 1.02 and an ${f_{nat}}$ of 0.80. Thus, Multi-LZerD attains medium-quality modelling of all protein-protein interfaces in the target with only minimal user-input constraints. The computation for all stages of docking for this target took less than 24 hours.

### Overall performance of LZerD in CAPRI in 2016–2018

In the recent CAPRI rounds 38–45 (2016–2018) there were seven targets which were modelled by using LZerD (17). There were 2 targets (T123 and T124) where individual input subunits were not well-modeled, with backbone RMSDs exceeding 10 Å. For the rest of five targets, four of them were correctly modelled with 2 of medium and 2 of acceptable CAPRI quality (11). The correctly modeled targets had subunit RMSDs from 0.5 Å to 7.0 Å. Except for the subunit modelled at a 7.0 Å RMSD, the rest of the subunit RMSDs were 0.5–2.8 Å. Note that building a CAPRI acceptable docking model is possible with a subunit of a 7.0 Å RMSD because the CAPRI criteria are mainly concerned with accuracy at the interaction interface. The remaining incorrectly modeled target had subunit RMSDs from 0.9 to 2.4 Å. Therefore, as long as subunit models have an RMSD of about up to 3 Å, LZerD has a good chance to be able to build acceptable quality models. Based on the results from CAPRI, LZerD performance depends on quality of structure information of subunits of a given complex. Therefore, LZerD is suited for cases where high-resolution structure information or an accurate computational model is available for each subunit of the protein complex of interest.

### Future expansion of our webserver platform

The LZerD webserver currently makes the highly ranked pairwise and multiple protein-protein docking functionality of LZerD and Multi-LZerD conveniently available to end users, along with the ability to integrate biological information in the form of geometric constraints. The full LZerD suite will further include functionality e.g. for assembling complexes from disordered proteins (24) and predicting the assembly pathways of protein complexes (25). The stand-alone programs of these tools, LZerD, Multi-LZerD, IDP-LZerD, Path-LZerD and PI-LZerD (26) are available at http://kiharalab.org/proteindocking/ (27). Future developments of the LZerD webserver will make more of the LZerD suite available through the same convenient and accessible interface.

## References

[1] Aderinwale T. , Christoffer C.W., Sarkar D., Alnabati E., Kihara D. Computational structure modeling for diverse categories of macromolecular interactions. Curr. Opin. Struct. Biol. 2020; 64:1–8. 32599506 10.1016/j.sbi.2020.05.017 PMC7665979

[2] Venkatraman V. , Yang Y.D., Sael L., Kihara D. Protein-protein docking using region-based 3D Zernike descriptors. BMC Bioinformatics. 2009; 10:407. 20003235 10.1186/1471-2105-10-407 PMC2800122

[3] Esquivel-Rodriguez J. , Yang Y.D., Kihara D. Multi-LZerD: multiple protein docking for asymmetric complexes. Proteins. 2012; 80:1818–1833. 22488467 10.1002/prot.24079 PMC3370124

[4] Mintseris J. , Pierce B., Wiehe K., Anderson R., Chen R., Weng Z. Integrating statistical pair potentials into protein complex prediction. Proteins. 2007; 69:511–520. 17623839 10.1002/prot.21502

[5] Dominguez C. , Boelens R., Bonvin A.M. HADDOCK: a protein-protein docking approach based on biochemical or biophysical information. J. Am. Chem. Soc. 2003; 125:1731–1737. 12580598 10.1021/ja026939x

[6] Kozakov D. , Hall D.R., Xia B., Porter K.A., Padhorny D., Yueh C., Beglov D., Vajda S. The ClusPro web server for protein-protein docking. Nat. Protoc. 2017; 12:255–278. 28079879 10.1038/nprot.2016.169 PMC5540229

[7] Lyskov S. , Gray J.J. The RosettaDock server for local protein-protein docking. Nucleic Acids Res. 2008; 36:W233–W238. 18442991 10.1093/nar/gkn216 PMC2447798

[8] Ritchie D.W. , Venkatraman V. Ultra-fast FFT protein docking on graphics processors. Bioinformatics. 2010; 26:2398–2405. 20685958 10.1093/bioinformatics/btq444

[9] Torchala M. , Moal I.H., Chaleil R.A., Fernandez-Recio J., Bates P.A. SwarmDock: a server for flexible protein-protein docking. Bioinformatics. 2013; 29:807–809. 23343604 10.1093/bioinformatics/btt038

[10] de Vries S. , Zacharias M. Flexible docking and refinement with a coarse-grained protein model using ATTRACT. Proteins. 2013; 81:2167–2174. 23996217 10.1002/prot.24400

[11] Lensink M.F. , Nadzirin N., Velankar S., Wodak S.J. Modeling protein-protein, protein-peptide, and protein-oligosaccharide complexes: CAPRI 7th edition. Proteins. 2020; 88:916–938. 31886916 10.1002/prot.25870

[12] Lensink M.F. , Brysbaert G., Nadzirin N., Velankar S., Chaleil R.A.G., Gerguri T., Bates P.A., Laine E., Carbone A., Grudinin S. et al. Blind prediction of homo- and hetero-protein complexes: The CASP13-CAPRI experiment. Proteins. 2019; 87:1200–1221. 31612567 10.1002/prot.25838 PMC7274794

[13] Novotni M. , Klein R. Proceedings of the Eighth ACM Symposium on Solid Modeling and Applications. 2003; 216–225.

[14] Zhou H. , Zhou Y. Distance-scaled, finite ideal-gas reference state improves structure-derived potentials of mean force for structure selection and stability prediction. Protein Sci. 2002; 11:2714–2726. 12381853 10.1110/ps.0217002 PMC2373736

[15] Zhou H. , Skolnick J. GOAP: a generalized orientation-dependent, all-atom statistical potential for protein structure prediction. Biophys. J. 2011; 101:2043–2052. 22004759 10.1016/j.bpj.2011.09.012 PMC3192975

[16] Huang S.Y. , Zou X. Statistical mechanics-based method to extract atomic distance-dependent potentials from protein structures. Proteins. 2011; 79:2648–2661. 21732421 10.1002/prot.23086 PMC11108592

[17] Christoffer C. , Terashi G., Shin W.H., Aderinwale T., Maddhuri Venkata Subramaniya S.R., Peterson L., Verburgt J., Kihara D. Performance and enhancement of the LZerD protein assembly pipeline in CAPRI 38–46. Proteins. 2020; 88:948–961. 31697428 10.1002/prot.25850 PMC7685511

[18] La D. , Kong M., Hoffman W., Choi Y.I., Kihara D. Predicting permanent and transient protein-protein interfaces. Proteins. 2013; 81:805–818. 23239312 10.1002/prot.24235 PMC4084939

[19] La D. , Kihara D. A novel method for protein-protein interaction site prediction using phylogenetic substitution models. Proteins. 2012; 80:126–141. 21989996 10.1002/prot.23169 PMC3240730

[20] Webb B. , Sali A. Protein structure modeling with MODELLER. Methods Mol. Biol. 2017; 1654:39–54. 28986782 10.1007/978-1-4939-7231-9_4

[21] Moonens K. , Hamway Y., Neddermann M., Reschke M., Tegtmeyer N., Kruse T., Kammerer R., Mejias-Luque R., Singer B.B., Backert S. et al. *Helicobacter pylori* adhesin HopQ disrupts trans dimerization in human CEACAMs. EMBO J. 2018; 37:e98665. 29858229 10.15252/embj.201798665 PMC6028033

[22] Perozzo R. , Kuo M., Sidhu A., Valiyaveettil J.T., Bittman R., Jacobs W.R. Jr, Fidock D.A., Sacchettini J.C. Structural elucidation of the specificity of the antibacterial agent triclosan for malarial enoyl acyl carrier protein reductase. J. Biol. Chem. 2002; 277:13106–13114. 11792710 10.1074/jbc.M112000200

[23] Arnold K. , Bordoli L., Kopp J., Schwede T. The SWISS-MODEL workspace: a web-based environment for protein structure homology modelling. Bioinformatics. 2006; 22:195–201. 16301204 10.1093/bioinformatics/bti770

[24] Peterson L.X. , Roy A., Christoffer C., Terashi G., Kihara D. Modeling disordered protein interactions from biophysical principles. PLoS Comput. Biol. 2017; 13:e1005485. 28394890 10.1371/journal.pcbi.1005485 PMC5402988

[25] Peterson L.X. , Togawa Y., Esquivel-Rodriguez J., Terashi G., Christoffer C., Roy A., Shin W.H., Kihara D. Modeling the assembly order of multimeric heteroprotein complexes. PLoS Comput. Biol. 2018; 14:e1005937. 29329283 10.1371/journal.pcbi.1005937 PMC5785014

[26] Li B. , Kihara D. Protein docking prediction using predicted protein-protein interface. BMC Bioinformatics. 2012; 13:7. 22233443 10.1186/1471-2105-13-7 PMC3287255

[27] Esquivel-Rodriguez J. , Filos-Gonzalez V., Li B., Kihara D. Pairwise and multimeric protein-protein docking using the LZerD program suite. Methods Mol. Biol. 2014; 1137:209–234. 24573484 10.1007/978-1-4939-0366-5_15

